# Plant Functional Diversity Is Primarily Influenced by Exchangeable Cation Deposition in a Saline‐Alkaline Coal‐Mining Region in Northwestern China

**DOI:** 10.1002/ece3.72862

**Published:** 2026-01-26

**Authors:** Chunhuan Li, Hailong Yu, Bing Li, Shengyi Huang, Juying Huang

**Affiliations:** ^1^ School of Ecology and Environment Ningxia University Yinchuan China; ^2^ The Key Laboratory for Silviculture and Conservation of Ministry of Education, College of Forestry Beijing Forestry University Beijing China; ^3^ School of Geography and Planning Ningxia University Yinchuan China; ^4^ School of Forestry and Prataculture Ningxia University Yinchuan China; ^5^ Research Institute of Subtropical Forestry Chinese Academy of Forestry Fuyang China

**Keywords:** biodiversity, ecosystem stability, industrial acid emissions, leaf functional trait, saline‐alkaline soil

## Abstract

Artificial sulfur (S) and nitrogen (N) addition experiments often fail to accurately simulate acid deposition in terms of type, composition, intensity, frequency, and duration, potentially leading to biased estimates of deposition impact on plant diversity. Consequently, studying plant diversity patterns around acid emission sources provides a more reliable alternative. Yet, this approach remains understudied in field research, particularly in saline‐alkaline regions where high soil buffering capacity may attenuate plant sensitivity to acid deposition. Therefore, we investigated plant functional diversity (PFD) and analyzed its influencing factors in a desert coal‐mining region in northwestern China characterized by high pH, abundant CaCO_3_ content in soils, and increasing acid deposition. The plant communities were characterized by high leaf thickness, low specific leaf area, and limited leaf total carbon (C) and N concentrations, indicating the preference of the plant communities for a slow investment‐returning ecological strategy in the study region. In this context, leaf traits (e.g., thickness and total C and N concentrations), rather than PFD, played a major role in stabilizing plant communities. The intensity of S and N deposition had no effect on PFD. In contrast, exchangeable cation (BC) deposition directly reduced the functional richness, functional dispersion, and the Rao's indices, possibly by exacerbating soil salinity and alkalinity in the study region. Our findings indicate that PFD is mainly influenced by BC deposition in saline‐alkaline coal‐mining regions. Therefore, the potential risk of BC deposition, which accompanies acid deposition, posed on plant diversity should not be overlooked in these regions.

## Graphical abstract

Although sulfur and nitrogen deposition had limited effects on plant functional diversity, the study found that leaf traits played a key role in maintaining plant community stability under acid deposition conditions.

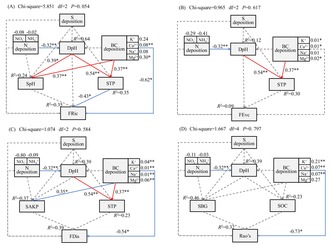

## 1 Introduction

Since the onset of the Industrial Revolution, human activity has produced substantial amounts of SO_2_ and NO_X_, leading to increasingly severe acid deposition worldwide (IPCC 2021). While moderate deposition of sulfur (S) and nitrogen (N) can benefit plant growth (Wang et al. 2021; Zhang, Wang, et al. 2024; Zhang, Xiang, et al. 2024), excessive inputs of these elements may result in soil acidification and biodiversity loss (Jung et al. 2018; Zarfos et al. 2019), ultimately impairing ecosystem services. Complicating its assessment, acid deposition is not a simple chemical input but a complex mixture of multiple ions, including SO_4_^2−^, NO_3_^−^, NH_4_^+^, H^+^, Cl^−^, F^−^, K^+^, Ca^2+^, Na^+^, and Mg^2+^, which together exert complicated effects on plants. Moreover, as a persistent environmental stressor, it causes chronic disturbances to ecosystems over long periods, with deposition patterns continuously evolving in terms of dry‐to‐wet ratio and S—N composition (Kopriva et al. 2019; Zhu et al. 2020). To evaluate the ecological effects, numerous field experiments have attempted to simulate acid deposition by adding S and N fertilizers to ecosystems (e.g., Feng et al. 2024; Hu et al. 2022; Liu et al. 2022; Ma et al. 2021; Niu et al. 2018; Xu et al. 2018; Zhang et al. 2022; Zhang, Wang, et al. 2024; Zhang, Xiang, et al. 2024). However, these studies are inherently limited in their capacity to replicate realistic deposition conditions in terms of ion composition, intensity, frequency, and duration. As a result, their findings may not accurately reflect plant responses under realistic conditions, potentially leading to an overestimation of deposition's negative impacts (Cao et al. 2020; Jung et al. 2018; Ke et al. 2023; Niu et al. 2018). Given these limitations in experimental simulations, there is a clear need to complement current approaches with direct observational studies conducted around acid emission sources.

Species diversity and functional diversity are key components of biodiversity. The traditional view is that species diversity dominates plant community stability (Chen, Jiang, et al. 2021). Therefore, acid deposition‐induced loss of plant species may lead to the degradation of ecosystem function (e.g., stability). However, recent studies have challenged this view and have suggested that the dominant mechanism of community stability in response to acid addition varies depending on the level of acid addition and duration of acid treatment (Hu et al. 2022; Ma et al. 2021; Niu et al. 2018). As a new perspective for predicting ecosystem function, functional diversity integrates plant functional strategies and is therefore considered to be closely related to the stability and productivity of an ecosystem (Xu et al. 2018). Evidence from forests, peatlands, and grasslands has shown that, compared to species diversity, functional diversity is more sensitive to environmental stress or perturbations (e.g., increasing acid deposition) and provides a more accurate representation of changes in ecosystem function (Bongers et al. 2021; Laine et al. 2021; Xu et al. 2018). An integrated analysis based on 258 field studies from temperate, subtropical, and tropical forests and grasslands further reported that functional diversity is more important than species diversity for ecosystem function (van der Plas 2019). This raises the question of whether functional diversity also plays a determining role in maintaining desert stability, particularly in areas experiencing increased acid deposition.

The extent and direction of the effect of S and N deposition on plant diversity depends on the initial soil pH (Chen et al. 2023; Midolo et al. 2019; Guo et al. 2016). The loss of plant diversity resulting from acid deposition has been verified in acidic and neutral soils (Jung et al. 2018; Li, Gan, et al. 2022; Zhang et al. 2022). Compared with these soil types, saline‐alkaline soils typically exhibit stronger acid buffering performance owing to their abundant carbonate content (Wei et al. 2022; Zhang et al. 2022). Therefore, acid deposition may have different effects on plant communities growing in saline‐alkaline soils. Previous simulation studies have shown that experimental S input can regulate the salinity and alkalinity of soils (Zhang et al. 2022) and improve saline‐alkaline environments, thereby increasing plant diversity (Tibbett et al. 2019). Similarly, artificial N addition reduced plant diversity in slightly saline‐alkaline soils but did not alter it in moderate (Gao et al. 2019) and severe (Xu et al. 2023) soils. Moreover, acid addition alters the nutrient availability in soils and disrupts the balance of resource competition among plant species. Species exhibiting favorable leaf functional traits can outcompete other species (Sun et al. 2022) and even exclude them from the ecosystem (Wilcots et al. 2021). However, additional research is required to determine how natural acid deposition affects the structure of plant communities in habitats with high soil salinity and alkalinity and whether this effect will lead to the loss of plant species at these sites.

Desert regions, which account for a large area of northwestern China, are an important ecological barrier to local socioeconomic development (Cao et al. 2022). The region is typically characterized by prevalent saline‐alkaline soils due to geographical and meteorological conditions (e.g., high latitude, low precipitation, and strong evaporation), which determine its high abiotic carbon (C) fixation potential and inorganic C sink function. Although the desert region is sparsely vegetated, it hosts many endemic species that have developed unique adaptive characteristics in terms of morphology, physiology, and ecology to maintain local ecosystem functions (Wei et al. 2013; Maestre et al. 2021). While it is well established that the effects of acid deposition on plant diversity vary with soil pH, research has predominantly focused on humid to semi‐arid regions. In contrast, studies on moderately to severely saline‐alkaline soils in arid zones remain scarce. The saline‐alkaline soils in arid regions (typically rich in SO_4_^2−^/Cl^−^ salts with Na_2_CO_3_/NaHCO_3_) are fundamentally different from those in humid to semi‐arid regions due to distinct formation processes (Vargas et al. 2018). These inherent compounds can interact uniquely with atmospheric deposits (e.g., N), potentially and collectively shaping plant diversity (Gao et al. 2019). This emphasizes the necessity of studying plant diversity in saline‐alkaline deserts.

The Ningdong Energy and Chemical Industry Base (hereafter referred to as Ningdong Base), located in the desert region of northwestern China, is an important component of China's Energy Golden Triangle. Although the current estimated acid deposition at Ningdong Base is lower than the national average, several risk factors, such as the low critical load of N deposition in soils (Zhao et al. 2021), the coupling of S and N deposition (Gao et al. 2018), and the cumulative nature of acid deposition over time (Cao et al. 2021), collectively highlight the necessity of accurately evaluating plant functional diversity (PFD) and its key drivers under the current acid deposition rate in this region. Therefore, we aimed to determine (1) which leaf functional traits plants develop to adapt to desert environments surrounding industrial acid emission sources, (2) whether PFD is closely related to plant community stability in a saline‐alkaline desert mining region, and (3) what the primary drivers shaping PFD in saline‐alkaline coal‐mining areas experiencing increased acid deposition are. The results of this study serve as a solid foundation for the sustainable conservation and restoration of deserts around industrial acid emission sources and provide a new approach for scientifically assessing the effects of acid deposition under various soil pH conditions.

## 2 Materials and Methods

### 2.1 Site Description

**FIGURE 1:**
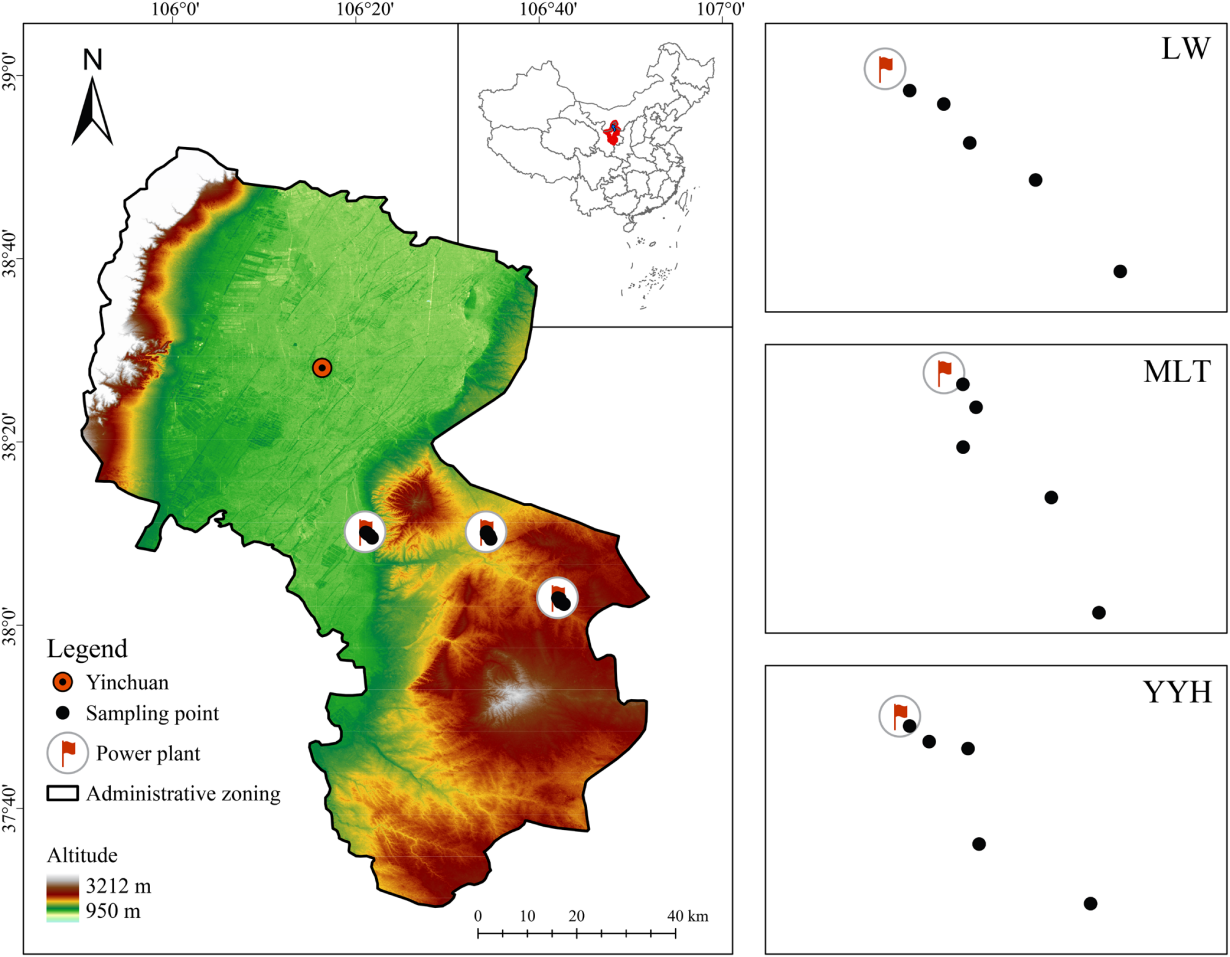
The geographical location of monitoring sites in the study region. LW, Lingwu power plant; MLT, Maliantai power plant; YYH, Yuanyanghu power plant.

The field experiment was conducted at Ningdong Base from July 2019 to December 2021. Ningdong Base, located in the middle of Ningxia in northwestern China (37°35′–38°21′ N, 106°11′–106°52′ E) (Figure 1), is characterized by a typical mid‐temperate continental climate with large temperature variation between day and night. The annual mean temperature for the region is 13.9°C. The coldest month is January, with an average temperature of −5.0°C, while July is the hottest month, with an average temperature of 25.5°C. From 2013 to 2023, the annual mean evaporation was 1633.1 mm, while the annual mean precipitation was 194.9 mm (Figure S1). During the 2019–2021 periods, the annual mean evaporation (1641.9, 1645.9, and 1625.2 mm) exhibited 10.0% interannual variability, whereas the corresponding precipitation (161.0, 192.9, and 192.3 mm) was markedly more stable, with only 0.7% variability. Data were sourced from the climate database of the China Meteorological Data Sharing Service System (https://data.cma.cn/).

The major soil types were sierozem, solonchak, solonetz, and aridisol. The soils contained 0.53–11.34 g·kg^−1^ of organic C content (SOC), 0.05–0.46 g·kg^−1^ of total N content (STN), and 0.17–0.46 g·kg^−1^ of total phosphorus content (STP). The cation exchange capacity was 7.40–9.20 cmol·kg^−1^ and the pH was 8.91 in the topsoil layer (Li, Wang, et al. 2022). The vegetation type was desert, composed of *Artemisia scoparia* Waldst. & Kit., *Stipa capillata* L., *Phragmites australis* (Cav.) Trin. ex Steud, *Artemisia desertorum* Spreng, *Tribulus terrestris* L., *Alhagi camelorum* Fisch., *Ammopiptanthus mongolicus* (Maxim. ex Kom.) Cheng, *Calligonum mongolicum* Turcz., and other annual and perennial drought‐resistant herbs and shrubs. With the rapid development of Ningdong Base, the surface soil and vegetation have been continuously destroyed owing to inadequate protection, resulting in further degradation of the local ecological environment.

### 2.2 Monitoring Site Selection

The monitoring site consisted of the area surrounding three power plants at Ningdong Base (Figure 1). The three power plants differed in their unit sizes: 2 × 330 MW for the Maliantai power plant, 2 × 600 MW plus 2 × 1000 MW for the Lingwu power plant, and 2 × 660 MW plus 2 × 1100 MW for the Yuanyanghu power plant. The vegetation surrounding the three power plants was sparse because of the geographic and climatic conditions, climate change, and disturbance from human activity.

Given that northwesterly winds prevail in the study area and that the maximum landing concentration of air pollutants occurs 1000–2000 m downwind of coal‐fired power plants (Luo et al. 2018; Tong 2016), southeast was chosen as the sampling direction and 0, 500, 1000, 1500, and 2000 m away from the enclosure wall of each selected power plant were chosen as the sampling distances (Figure 1). At each sampling distance from every power plant, three 10 × 10 m sampling points were set up in a representative fan‐shaped area (flat terrain, uniform vegetation distribution, and no tall trees within 100 m) for plant, bulk deposition, and soil sampling, resulting in a total of 15 points per power plant. The distance between two sampling points exceeded 10 m. According to a field survey conducted during 2018–2019, the selected sampling points were far from other industrial emission sources and human activity zones (e.g., villages, farmlands, pastures, and roads), ensuring the minimization of contamination from non‐coal‐fired power plant sources.

More details on the selection criteria for the sampling site, direction, distance, and points are provided by Li, Wang, et al. (2022).

#### 2.2.1 Plant Functional Diversity and Community Stability Calculation

To further eliminate spatial heterogeneity, three random 1 × 1 m quadrats were established at each sampling point for the plant composition survey in July 2020. With three points per distance, this resulted in a total of nine quadrats per sampling distance. During the peak vegetation growth period (early August) of 2020 and 2021, 10–15 healthy individuals of each species in the quadrats were selected for the measurement of specific leaf area (SLA), dry‐matter content (LDMC), chlorophyll content (LC), thickness (LT), density (LD), total C concentration (LTC), total N concentration (LTN), and total P concentration (LTP) of leaves. PFD (Functional richness index, FRic; Functional evenness index, FEve; Functional dispersion index, FDis; Rao's index, Rao's) was calculated based on the formulas in Table 1, with reference to Mason et al. (2005), Mouchet et al. (2010), and Villéger et al. (2008).

**TABLE 1:** Calculation of plant functional diversity.

Index	Calculation method	Terms meaning
Community‐weighted mean (CWM)	$\mathrm{CWM}=\sum_{i=1}^{n}P_{i}\cdot {\text{trait}}_{i}$	*trait*_*i*_ is the trait value of species *i*, while P_i_ is the relative abundance of species *i*.
Functional richness (*FR*_*ic*_)	${\mathrm{FR}}_{\mathrm{ic}}={\mathrm{SF}}_{\mathrm{ic}}/\mathrm{Rc}$	*SF*_*ic*_ is the ecological niche space occupied by the community, while *Rc* is that occupied by trait *C* in the community.
Functional evenness (*FE*_*ve*_)	${\mathrm{FE}}_{\mathrm{ve}}=\left(\sum_{i=1}^{s-1}\min\;\left({\mathrm{PEW}}_{I},\frac{1}{s-1}\right)-\frac{1}{s-1}\right)/\left(1-\frac{1}{s-1}\right)$ ${\mathrm{PEW}}_{i}=\frac{\text{EWbl}}{\sum_{b-1}^{s-1}\text{EWbl}}$ ${\mathrm{EW}}_{\mathrm{bl}}=\frac{d_{\mathrm{ij}}}{P_{i}+P_{j}}$	*S* is the species number in the community, while *P*_*i*_ and *P*_*j*_ are the relative abundance of species *i* and *j*, respectively.
Functional dispersion (*FD*_*is*_)	${\mathrm{FD}}_{\text{is}}=\sum a_{\mathrm{ij}}z_{j}/\sum a_{j}$ $C=\sum \left(a_{\mathrm{ij}}x_{\mathrm{ij}}\right)\sum a_{\mathrm{ij}}$	*a*_*j*_ is the abundance of species *j*. z_j_ is the distance of species *j* to the weighted centroid *C*. *X*_*ij*_ is the trait value of species *j* for trait *i*.
Rao's	${\mathrm{FD}}_{Q}=\sum_{i=1}^{s-1}\sum_{j=i+1}^{s}d_{\mathrm{ij}}p_{i}p_{j}$ $d_{\mathrm{ij}}=\sum_{t=1}^{T}{\left(X_{\mathrm{tj}}-X_{\mathrm{ti}}\right)}^{2}$	*d*_*ij*_ is the euclidian dissimilarity between the traits of each pair of species *i* and *j*, *X*_*ti*_ is the trait value of *i*_*th*_ species. *T* is the number of traits, *P*_*i*_ and *P*_*j*_ are the relative abundance of species *i* and *j*, respectively.

Community stability was expressed as the inverse of the coefficient of variation (CV) of species biomass, *ICV*, as follows:

$$\mathrm{ICV}=\frac{\mu }{\sigma }$$

where *μ* is the average biomass of each species in the quadrats and *σ* is the standard deviation of the biomass of each species. The variability of each species' biomass is smaller than that of the average biomass among species (Yang et al. 2011). Thus, a high *ICV* indicates high plant community stability.

### 2.3 Environmental Factor Measurement

A manual sampler combined with an alternative surface method was used to collect bulk deposition of rainfall and dustfall. Bulk deposition was collected once a week during months with frequent rainfall (from June to August) and once every 2 weeks during periods of scarce rainfall from July 2019 to December 2021. During bulk deposition collection, each sample was transferred to a washed polyethylene bottle and then quickly transported to the laboratory. Further details are provided by Li, Wang, et al. (2022).

In the laboratory, a portable acidimeter (S220, Mettler Toledo, Zurich, Switzerland) and portable conductance meter (S230, Mettler Toledo, Zurich, Switzerland) were used to determine the pH and electrical conductance, respectively. A continuous flow analyzer (Auto Analyzer 3, SEAL Analytical GmbH, Hanau, Germany) was used to determine the concentrations of SO_4_^2−^, NO_3_^−^, and NH_4_^+^. Inductively coupled plasma mass spectrometry (NexION 350X0303071402, Perkin Elmer, USA) was used to determine the concentrations of K^+^, Ca^2+^, Na^+^, and Mg^2+^. The sum of NO_3_^−^ and NH_4_^+^ was defined as the total inorganic N concentration. The annual mean deposition of each ion was calculated based on the nozzle area of the gauze and concentration of each ion (Li, Wang, et al. 2022).

Soil samples were collected from each of the three quadrats using a soil auger (5 cm diameter, 20 cm depth) and then combined into a single bulk sample per sampling point. To ensure timely measurements, the soil samples were immediately transported to the laboratory in an icebox. Available P concentration was then extracted using 0.5 M NaHCO_3_ and measured using the molybdenum blue method. Enzyme activity was measured through microplate fluorimetry using a full‐wavelength enzyme calibrator (M200 PRO, Tecan, Männedorf, Switzerland) at 365 nm excitation and 450 nm emission wavelengths. Meanwhile, Ca^2+^ and Mg^2+^ concentrations were determined using atomic absorption spectroscopy, whereas Na^+^ and K^+^ concentrations were analyzed using flame photometry following the methods described by Stutter et al. (2003). The pH, electrical conductance, NO_3_^−^ content, and NH_4_^+^ content were measured using the same methods as those used for each bulk deposition index. After the measurements were recorded, all soil samples were air‐dried and used for content analyses of organic C (K_2_MnO_4_ volume method), total N (Kjeldahl acid‐digestion method), and total P (HCLO_4_‐H_2_SO_4_ method).

### 2.4 Statistical Methods

For each index, the data at a given sampling distance was the mean value calculated from the three sampling points. Excel 2007 was used to compute the coefficient of variation (CV) (ratio of standard deviation to mean value) for each plant index. The degree of variation was determined based on the CV values. Specifically, a CV ranging from 0% to 15% indicates a weak variation, while CVs of 16%–35% and > 36% indicate moderate and large variations, respectively (Rogowski 1972). Origin 2018 and the ggplot2 package in R 4.0.3 were employed to generate partial plots showing the mean values and standard errors of the plant indices. To identify the strategy spectrum of plant growth, we conducted Principal Component Analysis (PCA) for the community‐weighted mean (CWM) of all leaf traits, as described by Maire et al. (2012). To investigate the relationships among plant community stability, PFD, and leaf traits, we used the ggplot2 package in R to perform single linear regression analyses. We used the MuMIn package in R for multiple regression modeling to assess the effects of bulk deposition and soil properties on the PFD indices. Furthermore, we conducted the Mantel Test using the linkET package in R and constructed a Structural Equation Model (SEM) using the lavaan package in R to develop the Prior Model (Figure S2, Online Resource 1). Subsequently, we eliminated nonsignificant paths from the Prior Model to create the best‐fit model. The selection criteria for the best‐fit model included nonsignificant *χ* ^2^ test results, the goodness‐of‐fit index, and the approximated root mean square error. Finally, we used the first axis of the PCA to quantify the model based on exchangeable cation (BC) deposition, N deposition, and soil BC content using the vegan package in R, as described by Oksanen et al. (2019).

## 3 Results

### 3.1 Variations in Plant Functional Diversity and Leaf Traits

**FIGURE 2:**
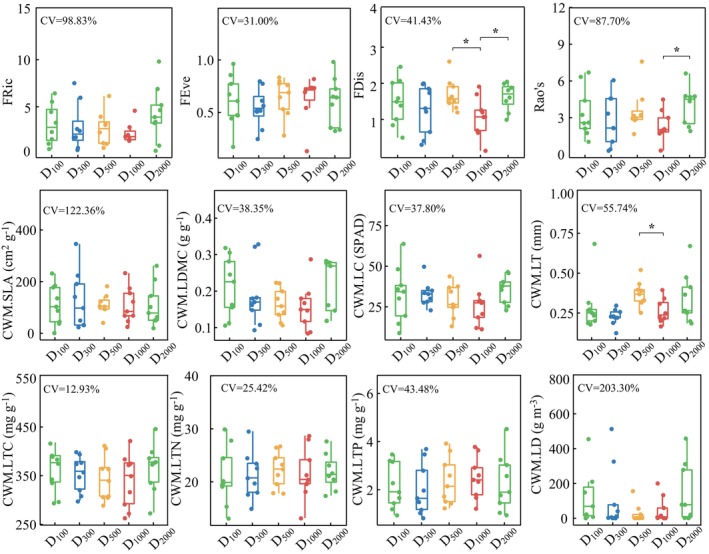
Changes in the indices of plant functional diversity and the community‐weighted means (CWMs) of leaf traits in the study region. “*” means adjusted *p* ≤ 0.05. No values shown when adjusted *p* > 0.05. *FD*_*is*_, functional dispersion index; *FE*_*ve*_, functional evenness index; *FR*_*ic*_, functional richness index; LC, leaf chlorophyll; LD, leaf density; LDMC, leaf dry‐matter content; LT, leaf thickness; LTC, leaf total C concentration; LTN, leaf total N concentration; LTP, leaf total P concentration; Rao's, Rao's index; SLA, specific leaf area.

Except for that of the CWM.LTC, the CV values of PFD indices and the CWMs of leaf traits generally exceeded 15.00% (Figure 2). Specifically, CWM.LTC showed a weak variation, ranging from 262.59 to 445.77 g·kg^−1^. Meanwhile, FEve and CWM.LTN showed moderate variations, ranging from 0.13 to 0.98 and from 13.05 to 46.70 g·kg^−1^, respectively. In contrast, FRic, FDis, Rao's, CWM.SLA, CWM.LDMC, CWM.LC, CWM.LT, CWM.LTP, and CWM.LD exhibited large variations.

There were no substantial differences among the five sampling distances (Figure 2). Specifically, FDis was lower at D_1000_ than at D_500_ and D_2000_ (*p* < 0.5), Rao's was lower at D_1000_ than at D_2000_ (*p* < 0.5), and CWM.LT was lower at D_1000_ than at D_500_ (*p* < 0.5).

**FIGURE 3:**
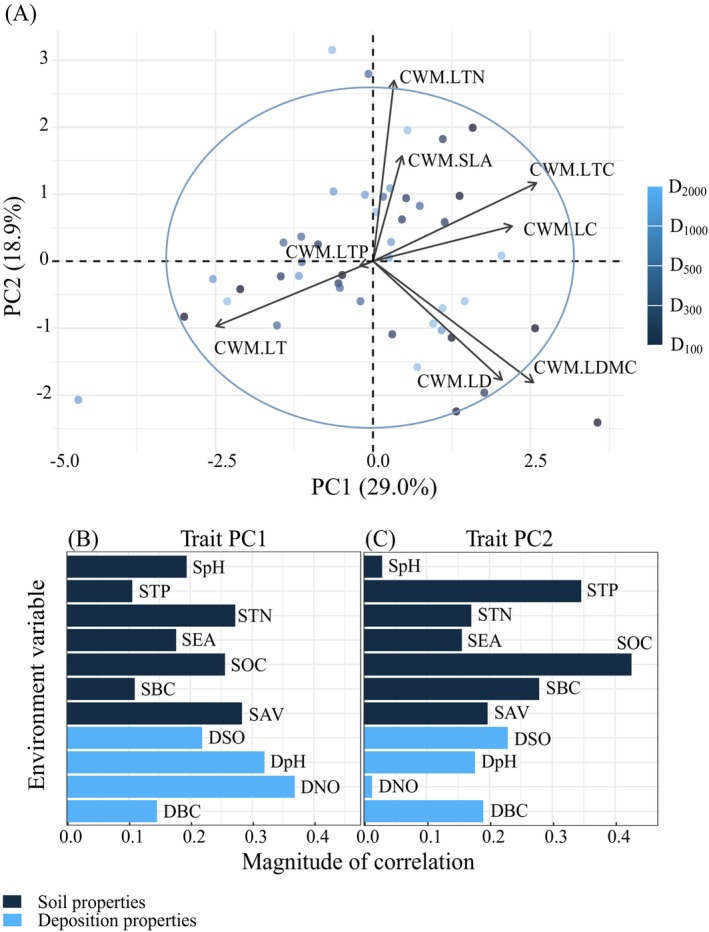
The first two principal component (PC) axes of the community‐weighted means (CWMs) for leaf traits and their correlation strengths with environmental properties. A, the first two principal trait axes (Trait PC1 and PC2). B and C, the correlations between the first two principal trait axes and environmental properties. LC, leaf chlorophyll; LD, leaf density; LDMC, leaf dry‐matter content; LT, leaf thickness; LTC, leaf total C concentration; LTN, leaf total N concentration; LTP, leaf total P concentration; SLA, specific leaf area.

The PCA results identified the main environmental drivers of multidimensional trait variation, with higher contributions from morphological traits (e.g., LTC, LDMC, and LT) in PC1 and photosynthetic traits (e.g., LTN and SLA) in PC2 (Figure 3a, Table 1). The strongest environmental drivers of trait covariation were identified by calculating the correlations between the individual environmental variables and PC1 and PC2 traits (Figure 3b,c). The results showed that PC1 was primarily correlated with DNO (*R* ^2^ = 0.369), whereas PC2 was strongly correlated with SOC (*R* ^2^ = 0.426).

### 3.2 Relationships Between Plant Community Stability and Functional Diversity and Leaf Traits

**FIGURE 4:**
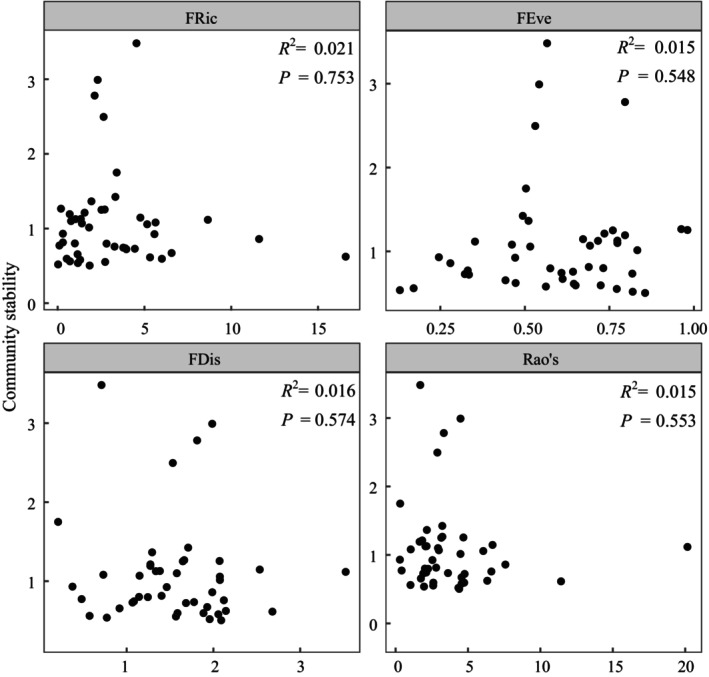
Relationships between the indices of plant functional diversity and plant community stability (*n* = 45). The shaded area represents a 95% confidence band. *FD*_*is*_, functional dispersion index; *FE*_*ve*_, functional evenness index; *FR*_*ic*_, functional richness index; Rao's, Rao's index.

**FIGURE 5:**
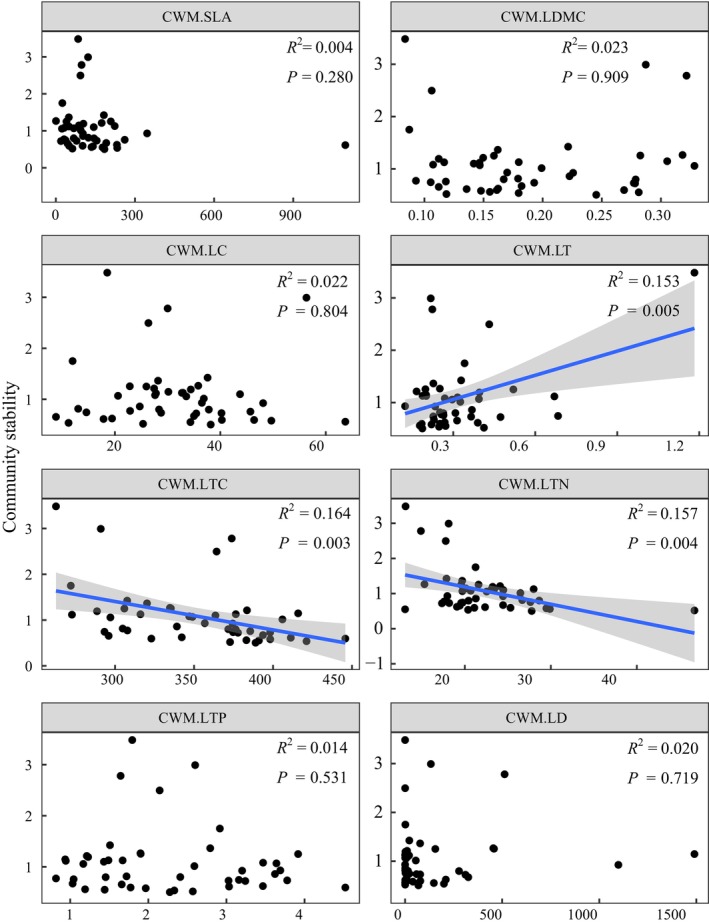
Relationships between the community‐weighted means (CWMs) of leaf traits and plant community stability (*n* = 45). The shaded area represents a 95% confidence band. LC, leaf chlorophyll; LD, leaf density; LDMC, leaf dry‐matter content; LT, leaf thickness; LTC, leaf total C concentration; LTN, leaf total N concentration; LTP, leaf total P concentration; SLA, specific leaf area.

There were no correlations between the PFD indices and community stability (*p* > 0.05) (Figure 4). In contrast, CWM.LT was positively correlated with community stability (*p* < 0.01), whereas CWM.LTC and CWM.LTN were negatively correlated with community stability (*p* < 0.01) (Figure 5).

### 3.3 Effects of Environmental Factors on Plant Functional Diversity

**FIGURE 6:**
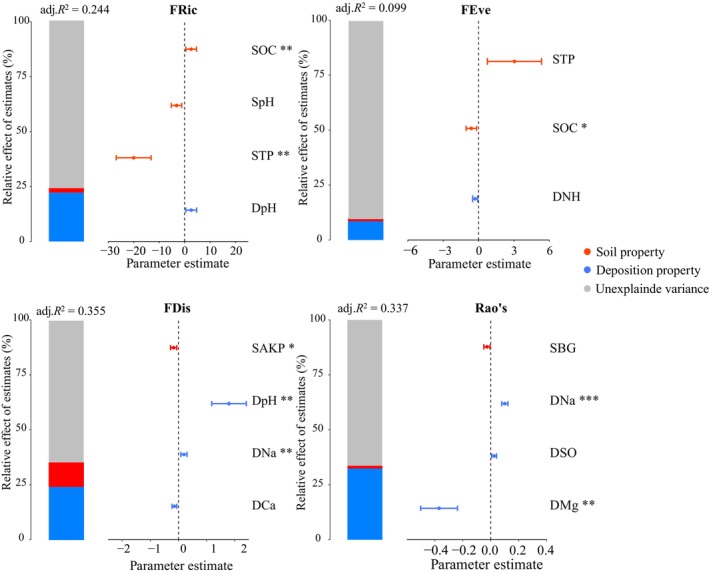
Relative effects of multiple predictors (deposition and soil properties) on the indices of plant functional diversity. The averaged parameter estimates (standardized regression coefficients) of the model predictors are shown with their associated 95% confidence intervals along with the relative importance of each predictor, expressed as the percentage of explained variance. The graph represents the best model selected based on the AICc. The relative effects of the predictors and their interactions can be calculated simply by determining the ratio between the parameter estimate of the predictor and the sum of all parameter estimates. This value is then expressed as a percentage. *, *p* < 0.05. **, *p* < 0.01. ***, *p* < 0.001. DCa, Ca^2+^ deposition; DMg, Mg^+^ deposition; DNa, Na^+^ deposition; DNH, NH_4_^+^ deposition; DpH, deposition pH; DSO, SO_4_^2−^ deposition; *FD*_*is*_, functional dispersion index; *FE*_*ve*_, functional evenness index; *FR*_*ic*_, functional richness index; Rao's, Rao's index; SAKP, soil alkaline phosphatase activity; SBG, soil β‐1,4‐Glucosidase activity; SOC, soil organic C content; SpH, soil pH; STP, soil total P content.

Compared with soil properties, deposition properties provided better explanations for the PFD indices, especially Na^+^ and Mg^+^ deposition. Among the soil properties, SOC, STP, and alkaline phosphatase activity were most closely correlated with the PFD indices (Figure 6).

**FIGURE 7:**
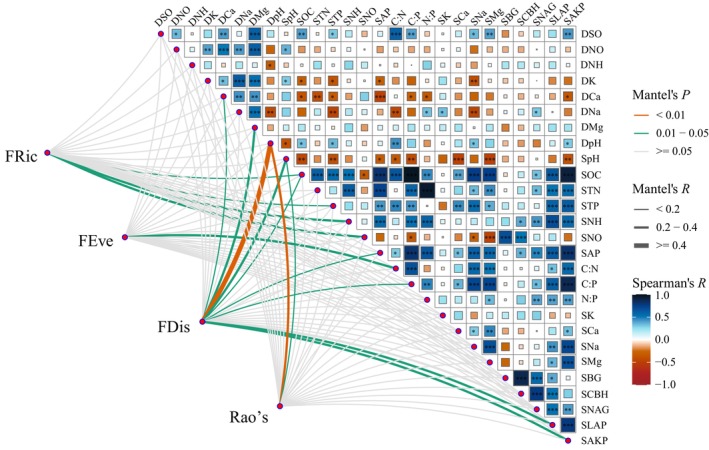
Correlations of deposition and soil properties with the indices of plant functional diversity. The color gradient represents the Spearman's correlation coefficient. FRic, Functional richness index. FEve, Functional evenness index. FDis, Functional dispersion index. Rao's, Rao's index. C:N, soil C:N; C:P, soil C:P; DCa, Ca^2+^ deposition; DK, K^+^ deposition; DMg, Mg^+^ deposition; DNa, Na^+^ deposition; DNH, NH_4_^+^ deposition; DNO, NO_3_^−^ deposition; DpH, deposition pH; DSO, SO_4_^2−^ deposition; N:P, soil N:P; SAKP, soil alkaline phosphatase activity; SAP, soil available P content; SBG, soil β‐1,4‐glucosidase activity; SCa, soil Ca^2+^ content; SCBH, soil cellobiose hydrolase activity; SK, soil K^+^ content; SLAP, soil leucine aminopeptidase activity; SMg, soil Mg^+^ content; SNa, soil Na^+^ content; SNAG, soil β‐1.4‐N‐acetylaminoglucosidase activity; SNH, soil NH_4_^+^‐N content; SNO, soil NO_3_^−^‐N content; SOC, soil organic C content; SpH, soil pH; STN, soil total N content; STP, soil total P content.

**FIGURE 8:**
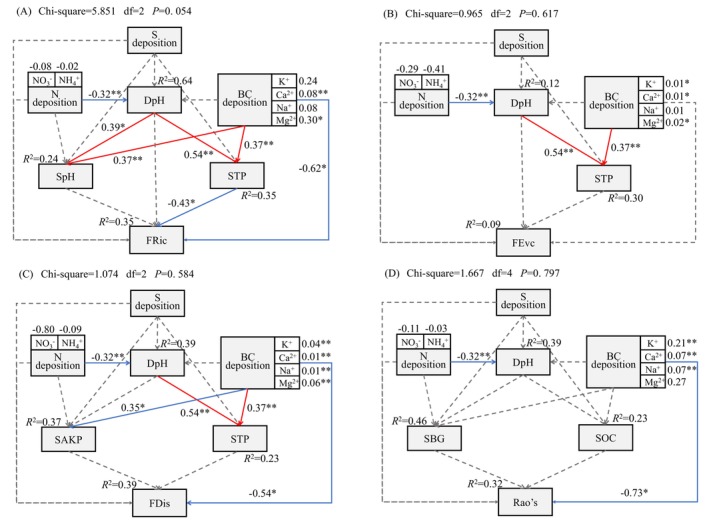
Influences of deposition and soil properties on the indices of plant functional diversity. A‐D represent the effects of sedimentation and soil properties on plant Functional Richness, Functional Evenness, Functional Dispersion, and Rao's Quadratic Entropy, respectively. The solid arrow indicates a significant path coefficient (*, *p* < 0.05. **, *p* < 0.01). The number on the solid arrow is the standardized path coefficient, which reflects the importance of each variable in the model. The dashed arrow indicates a nonsignificant path coefficient (*p* > 0.05). The number above the response variable is the proportion of variation explained by other variables (*R* ^2^). BC deposition, exchangeable cation deposition; DpH, deposition pH; FDis, functional dispersion index; FEve, functional evenness index; FRic, functional richness index; Rao's, Rao's index; SAKP, soil alkaline phosphatase activity; SBG, soil β‐1,4‐glucosidase activity; SOC, soil organic C content; SpH, soil pH; STP, soil total P content.

Based on the results shown in Figure S3 and Figures 6 and 7, we further determined the influence of environmental factors on the PFD indices using an SEM (Figure 8). We found that S deposition had no effect on any of the four indices (*p* > 0.05), whereas N deposition had an indirect negative effect on FRic through its impact on deposition pH and STP (*p* < 0.01). Meanwhile, BC deposition had direct negative effects on FRic, FDis, and Rao's (*p* < 0.05). It also indirectly and negatively affected FRic by positively affecting STP. Except for STP, soil properties did not affect any of the four PFD indices (*p* > 0.05).

## 4 Discussion

### 4.1 Preferred Ecological Strategy for Slow Investment‐Return of Desert Plants Around Industrial Acid Emission Sources

Based on a global leaf trait database, Wright et al. (2004) proposed the Leaf Economic Spectrum, which is defined as an ecological strategy used by plants to efficiently access resources and reasonably optimize the allocation of limited resources. In this study, the first component (PC1) distinguished plant communities based on leaf morphological traits (e.g., LTC, LMDC, and LT) related to resource conservation, whereas the second component (PC2) differentiated plant communities based on leaf photosynthetic traits (e.g., LTN and SLA) related to resource acquisition (Figure 3a, Table 2). The results clearly highlight the trade‐off between resource conservation and acquisition for desert plants in the study area, closely mirroring the findings at the species level (Diaz et al. 2016). The PCA results were predominantly located on the side associated with slow investment returning. Therefore, the results suggest that desert plants around industrial acid emission sources primarily adopt ecological strategies with a preference for conservative leaf traits to mitigate environmental risks, confirming the results of Maestre et al. (2021). Further analysis revealed that PC1 was primarily influenced by deposition properties rather than soil properties (Figure 3b), whereas PC2 exhibited the opposite pattern (Figure 3c). These results indicate that local plants prioritize reinforcing their physical support capabilities to withstand deposition risk, making the influence of soil properties relatively less pronounced.

**TABLE 2:** Principal component analysis (PCA) of the community‐weighted mean (CWMs) of leaf traits.

CWMs of leaf traits	PC1 scores	PC2 scores	Cumulative (%)
Dry‐matter content (CWM.LDMC)	0.474	0.418	47.9%
Chlorophyll (CWM.LC)	0.411	−0.121	
Total C concentration (CWM.LTC)	0.483	−0.270	
Thickness (CWM.LT)	−0.464	0.224	
Density (CWM.LD)	0.381	0.409	
Specific leaf area (CWM.SLA)	0.086	−0.363	
Total N concentration (CWM.LTN)	0.062	−0.623	
Total P concentration (CWM.LTP)	−0.039	0.016	

Plants in nutrient‐limited regions have evolved adaptive traits over extended periods to ensure survival. These adaptations often favor small, stress‐tolerant, and slow‐growing species characterized by specific leaf traits, including high nutrient conservation (e.g., low LTN and LTP) (Chen, Reed, et al. 2021) and thickness (e.g., low SLA and high LDMC) (Gong et al. 2017; Yang et al. 2018). In this study, the CWM values of total C, N, and P concentrations in leaves were lower than those in Chinese grasslands and forests (Liu et al. 2019; Zhang et al. 2019), indicating a relatively high nutrient conservation ability in the studied desert. Compared to national records (Zhang et al. 2019), these plants exhibited lower SLA and higher LDMC. However, their traits diverged from those in similar deserts far from acid emission sources (Liu et al. 2022; Yin et al. 2018), showing instead higher SLA and lower LDMC. SLA is widely recognized as an indicator of photosynthetic ability (Avalos 2023; Westoby et al. 2002), whereas traits such as LDMC and LTC reflect physical support structures (Mason and Donovan 2015; Wilson et al. 1999). Under harsh environmental conditions with constrained resource allocation, increased investment in traits like SLA may reduce allocation to traits such as LDMC and LTC, thereby maintaining an overall synergistic relationship among leaf traits (Ren et al. 2022; Shipley et al. 2006). Given the high acid deposition observed in the studied desert (Li, Wang, et al. 2022), the high SLA and low LDMC may indicate that acid deposition could enhance leaf photosynthesis, as reported by Liang et al. (2020). These findings confirm that desert plants actively regulate leaf traits to adapt to varying environmental conditions (Akram et al. 2020).

In most cases, neither the CWMs of leaf traits nor PFD exhibited clear trends along the sampling distance gradient (Figure 2). This absence of pattern can be attributed to two main factors. On one hand, after undergoing dust removal treatment, the particulate matter directly emitted from the coal‐fired power plants was characterized by relatively low concentrations and small particle sizes, facilitating long‐distance dispersion (Liang et al. 2019). As a result, its direct impact on plants near the emission sources may have been limited (Li, Wang, et al. 2022). On the other hand, the influence of acid deposition on plant diversity is known to vary significantly across different climate zones and ecosystem types (Bobbink et al. 2010; Han et al. 2019). Although the sampling points for each power plant were carefully selected to account for spatial heterogeneity, the three studied sites inherently differed in their meteorological conditions, soil properties, and vegetation composition (Li, Wang, et al. 2022). The interplay of these factors likely obscured any clear distance‐dependent patterns in these indices.

### 4.2 Leaf Traits, Rather Than Plant Functional Diversity, Predominantly Influence Plant Community Stability

Considerable evidence has shown that high biodiversity can stabilize the ecosystem functioning of grasslands over time (Liang et al. 2021; Ma et al. 2021; Su et al. 2023; Wu et al. 2023; Zhou et al. 2020). However, the relative importance of different aspects of biodiversity within the diversity‑stability relationship remains unclear (Craven et al. 2018). Previous studies have hypothesized that PFD plays a predominant role in enhancing the stability of plant communities because it integrates plant functional strategies (Hallett et al. 2017; van Klink et al. 2019). In this study, nonsignificant relationships were observed between PFD indices and community stability (Figure 4), suggesting that functional diversity may not be the primary factor driving changes in plant communities in natural ecosystems under global change (Miller et al. 2019). In contrast, the CWM values for leaf traits were more effective at characterizing community stability (Figure 5), with a positive linear relationship between LT and community stability but a negative linear relationship between LTN and community stability. These results emphasize that plant communities dominated by resource‐conserving ecological strategies, characterized by low leaf nutrient concentrations and robust leaf physical structures, tend to be more stable in resource‐limited environments (Conti et al. 2023; Majekova et al. 2014). This finding supports the theory of the “fast‐slow” functional trait economics spectrum, which posits that conservative plants possessing slow‐growing traits are more resistant to the stressors of barren environments (Reich 2014).

### 4.3 Predominant Role of Exchangeable Cation Deposition in Driving Plant Functional Diversity

Numerous field trials have investigated the response of plant species diversity to the addition of S and N (e.g., Chen et al. 2013; Feng et al. 2024; Jung et al. 2018; Liu et al. 2022; Tian et al. 2022; van der Plas et al. 2024; Zhang et al. 2022; Zhang, Wang, et al. 2024; Zhang, Xiang, et al. 2024). However, our understanding of how nutrient addition alters PFD is insufficient. Our results showed that S and N deposition, particularly S deposition, did not exert obvious effects on functional diversity and the CWM values of the main functional traits (Figure S3, Figure 7). Although the acid deposition in the study region was higher than the average level in northwestern China (Yu et al. 2019), S dominated the chemical composition of acid deposition (Li, Wang, et al. 2022), and S deposition was within the acceptable range (Duan et al. 2000; Li, Wang, et al. 2022). However, the studied soils were rich in CaCO_3_, thereby having a high capacity to buffer the effect of acid deposition (Duo et al. 2023). Therefore, the current intensity of S and N deposition did not significantly affect PFD. In contrast, BC deposition (especially Ca^2+^) had a significant negative effect on FRic, FDis, and Rao's (Figure 8). One possible reason for this is the strong positive correlation between BC deposition and soil Na^+^ content (Figure 7). This indicates that BC deposition aggravates soil salinization, which may hinder the growth of most local plants.

Evidence has shown that PFD is often weakly correlated to soil properties (Anderegg 2022; Maire et al. 2015). This study also showed that soil properties provided little explanation for variations in plant communities. However, STP had a negative impact on FRic (Figure 8), which reflects the extent to which species in a community occupy an ecological niche space, with a low FRic suggesting underutilization of the ecological niche space (Mason et al. 2005). According to the ecological niche dimension and diversity hypothesis, ecological niche differentiation driven by soil limiting factors is a critical mechanism for maintaining species coexistence within plant communities (Harpole and Tilman 2007). With increasing N deposition, plant nutrient limitation gradually shifts from N to P (Li, Wang, et al. 2022; Wang et al. 2022). Consequently, P‐deficient communities seek greater ecological niche differentiation to survive (Zhu et al. 2022). Conversely, an increase in soil P levels effectively alleviates P competition and reduces functional diversity (Mayfield and Levine 2010).

## 5 Conclusion

The plant communities in the study area mainly exhibited an ecological strategy characterized by a slower return on growth investment compared to that in other ecosystems (less limited by nutrients and water). However, they had higher leaf photosynthesis ability than plants in similar desert areas far from acid emission sources. Plant community stability showed a close correlation with leaf traits rather than with PFD, with a close positive linear relationship with LT and a negative linear relationship with LTC and LTN. Both S and N deposition had little effect on PFD. In contrast, BC deposition negatively affected FRic, FDis, and Rao's. Therefore, the effects of BC deposition on plant diversity should not be overlooked. These findings provide data support for a rational assessment of the impact of acid deposition on plant diversity surrounding sources of industrial acid emissions.

It should be noted, however, that the present study was conducted over a 3‐year period and only involved moderate saline‐alkaline soils. Although soil salinity and alkalinity may mitigate the adverse effects of acid deposition on plant diversity, chronic acidification elevates the risk of species loss (Chen et al. 2023). Emphasizing the long‐term and cumulative impacts of acid deposition in deserts would further underscore the significance of this work. Additionally, the effects of acid deposition on plant diversity interact with climate variability (Habas et al. 2025; Kazanski et al. 2021; Smith et al. 2016) and depend on soil pH (Chen et al. 2023; Midolo et al. 2019). Given the extreme weather event (e.g., temperature extreme and drought) driven by the climatic fluctuations, the moderate soil salinity‐alkalinity caused by abundant CaCO_3_ content, and the spatial heterogeneity induced by sparse vegetation in the studied desert, future studies should extend monitoring durations, include severely saline‐alkaline soils, and increase the number of sampling points. Such efforts would help clarify the drivers of PFD around acid emission sources, as well as the interactions between acid deposition and other potential environmental stressors across longer time scales and wider soil pH conditions.

## Author Contributions

**Chunhuan Li:** methodology (equal), writing – original draft (equal). **Hailong Yu:** formal analysis (equal), project administration (equal). **Bing Li:** formal analysis (equal), methodology (equal). **Shengyi Huang:** supervision, review and editing. **Juying Huang:** project administration (equal), writing – review and editing (equal).

## Funding

This work was supported by the Natural Science Foundation of Ningxia Province (2022AAC02012) and National Natural Science Foundation of China (32160277, 32371632).

## Conflicts of Interest

The authors declare no conflicts of interest.

## Supporting information


**Data S1:** ece372862‐sup‐0001‐supinfo.docx.

## Data Availability

The data underlying this article are available in GitHub at https://github.com/Chunhuan318/Functional‐Diversity.
