# Supplementary material for: Plant Functional Diversity Is Primarily Influenced by Exchangeable Cation Deposition in a Saline‐Alkaline Coal‐Mining Region in Northwestern China
Source: Ecol Evol. 2026 Jan 26;16(1):e72862. doi: 10.1002/ece3.72862 (PMC12834613; doi:10.1002/ece3.72862)
Supplement: Supplementary file 1 — Data S1: ece372862‐sup‐0001‐supinfo.docx. [file ECE3-16-e72862-s001.docx]

***Supplementary Materials***

**Plant functional diversity is primarily influenced by exchangeable cation deposition in a saline-alkaline coal-mining region in northwestern China**

**Authors:** Chunhuan Li^a,b^ , Hailong Yu^c^, Bing Li^d^, Juying Huang^a,*^

^a^ School of Ecology and Environment, Ningxia University, Yinchuan 750021, China;

^b^ Breeding Base for State Key Laboratory of Land Degradation and Ecological Restoration in northwestern China, Yinchuan 750021, China;

^c^ School of Geography and Planning, Ningxia University, Yinchuan 750021, China;

^d^ School of Forestry and Prataculture, Ningxia University, Yinchuan 750021, China

**^*^Correspondence author**:

Juying Huang (**Tel:** +86 13709586260; **E-mail:** [juyinghuang@163.com](mailto:juyinghuang@163.com))


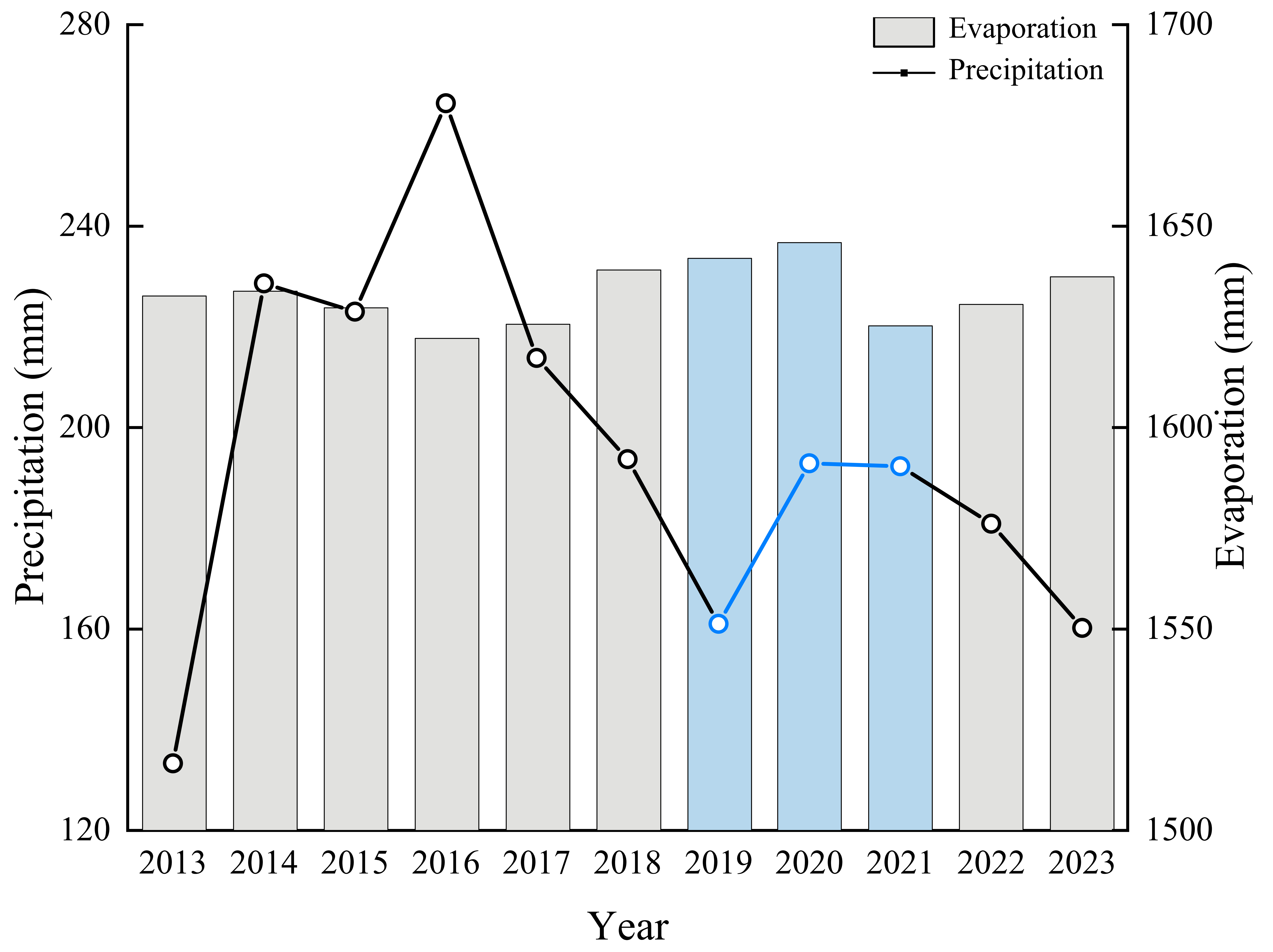


**Fig. S1** Annual average precipitation and evaporation in the study area from 2013 to 2023.


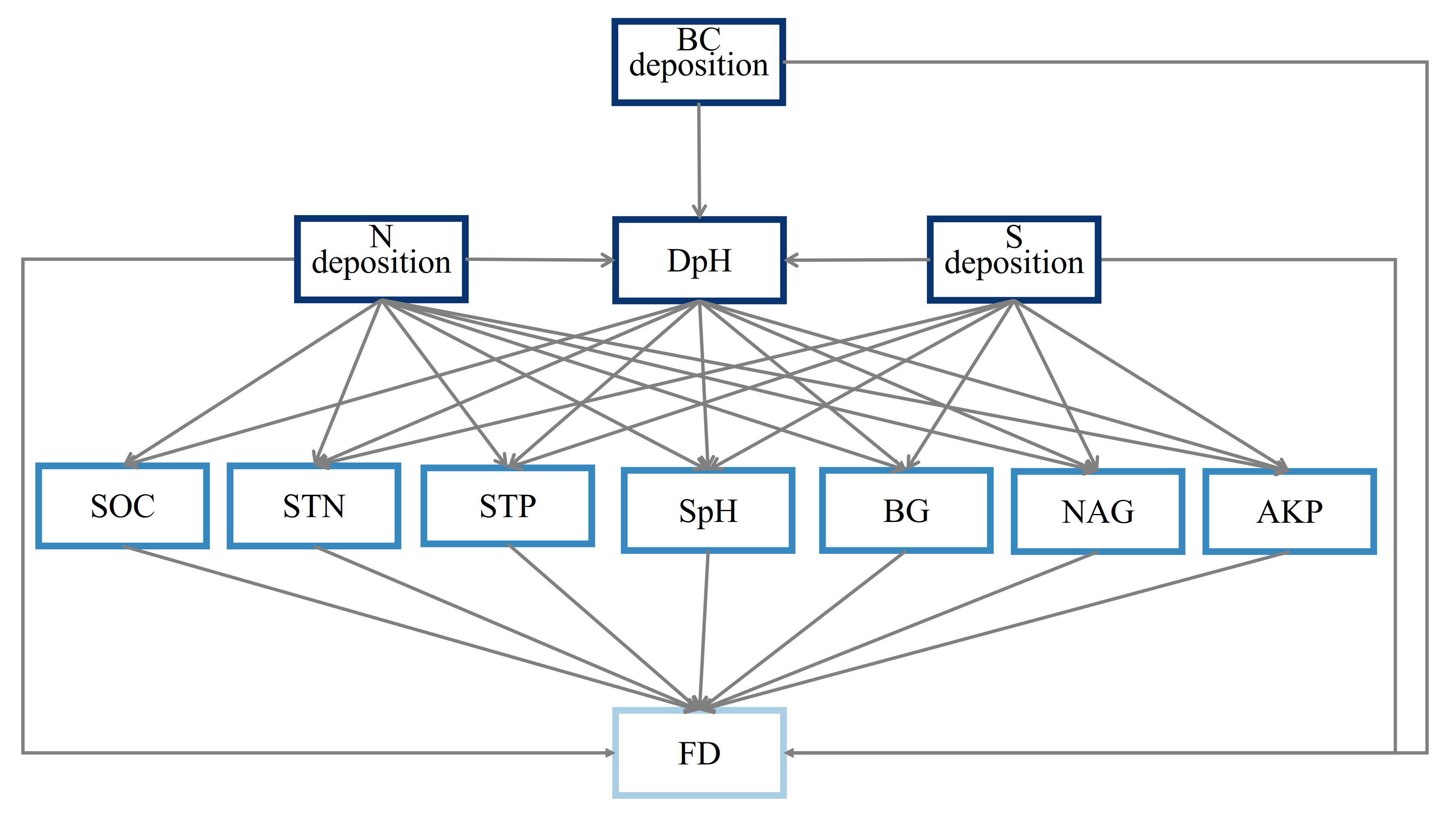


**Fig. S2** Priori model constructed from correlation tests and experience. See abbreviations in Table 2.


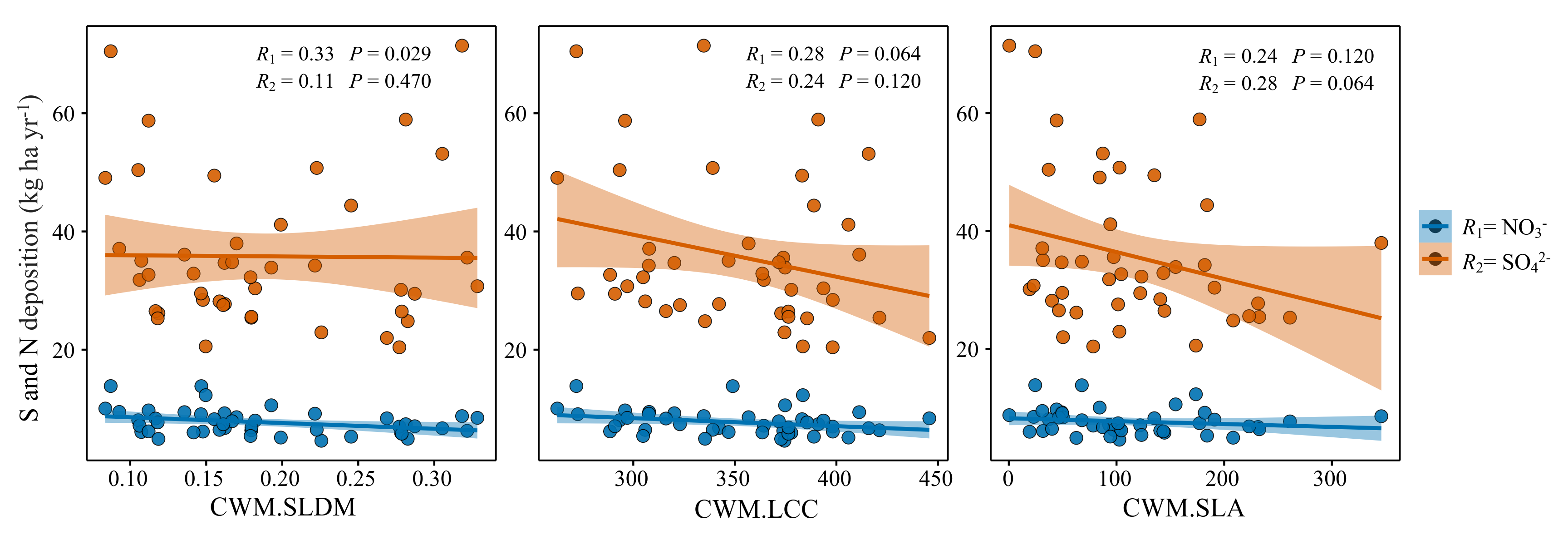


**Fig. S3** The relationship between functional traits and acid deposition. See abbreviations in Table 2.
